# Breathing space: deoxygenation of aquatic environments can drive differential ecological impacts across biological invasion stages

**DOI:** 10.1007/s10530-021-02542-3

**Published:** 2021-04-30

**Authors:** James W. E. Dickey, Neil E. Coughlan, Jaimie T. A. Dick, Vincent Médoc, Monica McCard, Peter R. Leavitt, Gérard Lacroix, Sarah Fiorini, Alexis Millot, Ross N. Cuthbert

**Affiliations:** 1 Institute for Global Food Security, School of Biological Sciences, Queen’s University Belfast, 19 Chlorine Gardens, Belfast, BT9 5DL Northern Ireland, UK; 2 Leibniz-Institute of Freshwater Ecology and Inland Fisheries (IGB), 12587 Berlin, Germany; 3 School of Biological, Earth and Environmental Sciences, University College Cork, Distillery Fields, North Mall, Cork, Ireland; 4 Equipe de Neuro-Ethologie Sensorielle (ENES), Centre de Recherche en Neurosciences de Lyon (CRNL), CNRS, INSERM, Université de Lyon/Saint-Etienne, Saint-Etienne, France; 5 Department of Biology, University of Regina, Regina, SK S4S 0A2 Canada; 6 iEES-Paris, Institut d’Ecologie et des Sciences de l’Environnement de Paris (IRD, Sorbonne Université, CNRS, INRA, UPEC, Université Paris Diderot), CC237 Paris, France; 7 Ecole Normale Supérieure, CNRS, Centre de Recherche en Écologie Expérimentale et Prédictive (CEREEP-Ecotron Ile-De-France), UMS 3194, PSL Research University, Saint-Pierre-lès-Nemours, France; 8 GEOMAR, Helmholtz-Zentrum für Ozeanforschung Kiel, 24105 Kiel, Germany

**Keywords:** Climate change, Functional responses, Hypoxia, Invasive alien species, *Neogobius melanostomus*

## Abstract

The influence of climate change on the ecological impacts of invasive alien species (IAS) remains understudied, with deoxygenation of aquatic environments often-overlooked as a consequence of climate change. Here, we therefore assessed how oxygen saturation affects the ecological impact of a predatory invasive fish, the Ponto-Caspian round goby (*Neogobius melanostomus*), relative to a co-occurring endangered European native analogue, the bullhead (*Cottus gobio*) experiencing decline in the presence of the IAS. In individual trials and mesocosms, we assessed the effect of high, medium and low (90%, 60% and 30%) oxygen saturation on: (1) functional responses (FRs) of the IAS and native, i.e. per capita feeding rates; (2) the impact on prey populations exerted; and (3) how combined impacts of both fishes change over invasion stages (Pre-invasion, Arrival, Replacement, Proliferation). Both species showed Type II potentially destabilising FRs, but at low oxygen saturation, the invader had a significantly higher feeding rate than the native. Relative Impact Potential, combining fish per capita effects and population abundances, revealed that low oxygen saturation exacerbates the high relative impact of the invader. The Relative Total Impact Potential (RTIP), modelling both consumer species’ impacts on prey populations in a system, was consistently higher at low oxygen saturation and especially high during invader Proliferation. In the mesocosm experiment, low oxygen lowered RTIP where both species were present, but again the IAS retained high relative impact during Replacement and Proliferation stages at low oxygen. We also found evidence of multiple predator effects, principally antagonism. We highlight the threat posed to native communities by IAS alongside climate-related stressors, but note that solutions may be available to remedy hypoxia and potentially mitigate impacts across invasion stages.

**Supplementary Information:**

The online version contains supplementary material available at 10.1007/s10530-021-02542-3.

## Introduction

The global spread of invasive alien species (IAS) has had wide-ranging consequences, such as negatively impacting ecosystem services, human health, economies and food security, while also contributing to the current sixth mass extinction (Ceballos et al. 2015; Turvey and Crees 2019; Cuthbert et al. 2021). With increasingly globalised transport networks creating novel pathways for IAS spread (Hulme 2009; Zieritz et al. 2016; Seebens et al. 2019), the numbers arriving in new locations will likely increase in the future (Seebens et al. 2018). These invasions will occur against a backdrop of changing climate and anthropogenic alterations of ecosystems, which may alter introduction success and ecological impacts of IAS (MacDougall and Turkington 2005; Rahel and Olden 2008; Zeng and Yeo 2018). This species-environment interaction is important as successful invaders commonly exhibit a greater tolerance to changes in the biophysical environment than do native taxa (Moyle and Light 1996; Grabowski et al. 2007). Understanding the interactions of IAS with environmental change is therefore crucial to both developing forecasts of IAS impacts and developing effective mitigation strategies (Hellmann et al. 2008).

There is growing empirical evidence that taxa from certain regions are predisposed to invasion success (Cuthbert et al. 2020; Paiva et al. 2018; Stern and Lee 2020), and high adaptability to new environments is an especially common trait for IAS originating from the Ponto-Caspian region, i.e. the Black, Azov and Caspian Sea areas (Ketelaars 2004; Gallardo and Aldridge 2015; Sturtevant et al. 2019). Many Ponto-Caspian species thrive at the expense of native taxa in areas of significant anthropogenic alteration (Den Hartog et al. 1992; Borza et al. 2017; Cerwenka et al. 2018; Bussmann and Burkhardt-Holm 2020) and, despite being from brackish conditions, many establish in high numbers in freshwater systems (Casties et al. 2016; Pauli et al. 2018). The development of improved methods for predicting which of such species are likely to establish, spread, proliferate and exert ecological impact is vital (Dick et al. 2017b); however, forecasts have thus far proven difficult due to the highly context-dependent nature of invasions and their impacts (Dick et al. 2017a). Further, management options for suppression or eradication of established invader populations are often complex, resource-intensive and expensive endeavours that require prioritisation based on cost–benefit analyses (Caffrey et al. 2011; Piria et al. 2017; Coughlan et al. 2018).

Of the myriad abiotic consequences of a changing climate, the implications of temperature increases have been most intensively studied (Dillon et al. 2010; Beaumont et al. 2011; Bellard et al. 2012). Although initially ignored, studies on aquatic systems now recognize the potential for effects of atmospheric warming, including declines in oxygen content of aquatic systems. Oxygen solubility in water is inversely related to water temperature, with higher water temperatures also increasing the biological oxygen demand of cold-blooded aquatic organisms, which can lead to oxygen demand exceeding supply (Ficke et al. 2007). Increased hypoxia has been reported in rivers (Blaszczak et al. 2019), lakes (Mallin et al. 2006; Jenny et al. 2016) and coastal waters, with rates increasing exponentially each year (Vaquer-Sunyer and Duarte 2008). Such thermally-induced hypoxia is further exacerbated by elevated levels of organic matter production and pollution (MacNeil et al. 2004), especially associated with urbanisation and intensive animal agriculture (Wen et al. 2017). Areas of decreased oxygen, known as ‘dead zones’ in marine studies, have a wide range of consequences for species, such as increased exposure to predators, suppressed immune responses and recruitment failure (Díaz et al. 2009). Crucially, some successful IAS have demonstrated a greater ability to survive and establish in such areas of low dissolved oxygen at the expense of natives (Jewett et al. 2005; Lagos et al. 2017). However, there is a crucial need for further studies assessing how such oxygen depleted zones and IAS act in concert (Norkko et al. 2012).

The round goby (*Neogobius melanostomus*) is a widespread predatory invasive fish from the Ponto-Caspian region that has spread across Europe and into the Great Lakes of North America. It has a broad diet, aggressive behaviour, numerous spawning events, parental care by males and a tendency to be larger than native trophic analogues (Dubs and Corkum 1996; Corkum et al. 2004; Bergstrom and Mensinger 2009). Impacts of round goby invasions include exclusion of native species (Hempel et al. 2016), trophic cascades ensuing from predation on invertebrates (Kipp and Ricciardi 2012), native fish population reductions and often total community replacements in European and North American waters (van Kessel et al. 2016); for example, the decline of mottled sculpin, *Cottus bairdi,* in Lake Michigan (Janssen and Jude 2001). Crucially, it is known to have a wide tolerance for abiotic stressors, such as temperature (Christensen et al. 2021), salinity (Behrens et al. 2017) and oxygen, with populations often experiencing seasonal hypoxia in their invasive range (Arend et al. 2011).

Here, we therefore assessed the potential ecological impact of *N. melanostomus* relative to an endangered trophically-analogous European native fish, the bullhead, *Cottus gobio*, at three oxygen saturation treatments representing high, medium and low saturations. Across its range, *C. gobio* has been threatened by a number of anthropogenic factors, including pollution and IAS (Utzinger et al. 2008; Lorenzoni et al. 2018). Further, this species has a co-evolutionary relationship with freshwater communities in western Europe, and hence serves as a baseline for comparison of ecological impacts with the invader (see Dick et al. 2017b).

**Fig. 1:**
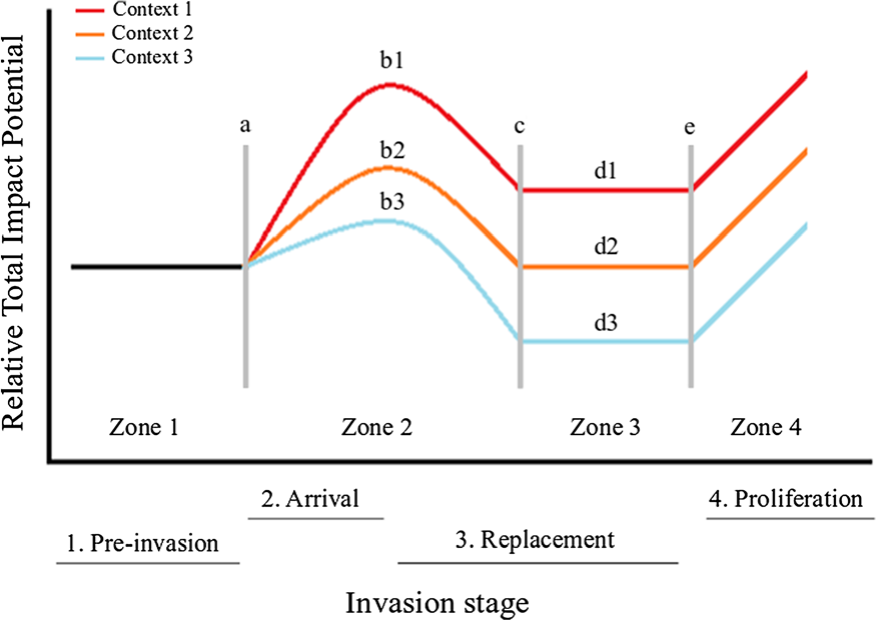
Conceptual spatio-temporal patterns of invasion impact across four invasion stages, under three hypothetical contexts (such as three different temperatures, salinities or dissolved oxygen levels). In Zone 1, the “Pre-invasion” baseline impact is driven by the native species before the invader arrives, and at point “a” the invasion takes place. In Zone 2, additional impact is exerted by the “Arrival” of the invader, that is, impact is driven by invader and native combined, up to a temporary impact peak, which might vary in magnitude, denoted “b1-b3” in Zone 2. Following these peaks, impact declines as the native undergoes “Replacement”, with the point of complete “replacement” denoted “c”. In Zone 3, with only the invader now present, the impact level may remain higher than the native species baseline. Further, in Zone 4, after point “e”, “Proliferation” of the invader may occur with consequent heightened impact

This paper uses three recently-developed methods to assess the effect of oxygen saturation on the ecological impacts of the two study species. First, the Comparative Functional Response method (CFR: Dick et al. 2014, 2017a) uses the classic metric of the functional response (FR: Solomon 1949; Holling 1959) to quantify how resource density influences resource consumption rates and thus ecological impact. This method also allows incorporation of a wide range of biotic and abiotic contexts, including habitat complexity (Cuthbert et al. 2019), temperature (Wasserman et al. 2018), higher order predators (Barrios-O’Neill et al. 2014), and parasites (Iltis et al. 2018), and has been shown to highlight known damaging invaders as having higher maximum feeding rates than trophic analogues (Dick et al. 2014). Here, we used CFR to assess the effects of oxygen regime on prey consumption by the invasive *N. melanostomus* and native *C. gobio*. With the tolerance of *N. melanostomus* to different abiotic stressors already outlined, and goby invasion success often facilitated by anthropogenic alteration of ecosystems, such as reduced dissolved oxygen levels (Cerwenka et al. 2018), we therefore hypothesise that the Ponto-Caspian invader will have a higher maximum feeding rate (FR asymptote) than the native across all oxygen treatments. Second, the Relative Impact Potential (RIP) metric combines the FR parameters (e.g. maximum feeding rate, 1/h) with a proxy for the Numerical Response (NR: e.g. consumer abundance or density) to increase overall predictive power of ecological impact (Dick et al. 2017b; Dickey et al. 2020). Third, we introduce the new Relative Total Impact Potential (RTIP) metric to assess the overall fish species’ impact (i.e. combined impact of IAS and native) on a system over four invasion stages: Pre-invasion, Arrival, Replacement and Proliferation (Fig. 1; see also Dickey et al. 2020). For example, across these invasion stages, the impact exerted by the invader may add to that already being exerted by the native, perhaps leading to a temporary, amplified impact on the system (Fig. 1). We assessed the differing impacts of the two fish species within each invasion stage in two ways: (1) using consumption data from our FR experiment with individual predators and combining those data with actual recorded field density data of the two fish species (from areas of one river at different stages of *N. melanostomus* invasion, and an uninvaded river for a “baseline” native species density), and; (2) a mesocosm experiment to simulate the four invasion stages, with multiple predators of both fish species, which is important to capture multiple predator effects (MPEs) that may be additive, synergistic or antagonistic (Mofu et al. 2019).

## Methods

### Animal collection and maintenance

Fish were sampled via electrofishing (*n* = 40 ind. species^−1^). The invasive round goby, *Neogobius melanostomus,* was collected on the 6th of October 2018 from the Moselle River at Koenigsmacker, Moselle, France (49°24′14.6″N 6°15′24.3″E), and the native bullhead, *C. gobio,* on the 4th of October 2018 from Ru du Dragon, Longueville, France (48°31′20.4″N 3°14′22.3″E), where *N. melanostomus* has not yet invaded. Fish selected for experiments were matched as closely as possible with respect to total length (TL mm ± SD: goby = 83.0 ± 5.45; bullhead = 80.4 ± 3.41) and mouth gape height (GH mm ± SD: goby = 7.12 ± 0.97; bullhead = 8.3 ± 0.62) to quantify species-specific differences unrelated to fish size and mouth gape. If anything, this may underestimate the ecological impacts of the invader that tends to grow larger than the native (max. recorded total length of *N. melanostomus* 30 cm; max. recorded total length of *C. gobio* 18 cm: Froese and Pauli 2020). Fish were transported in continuously aerated source water and housed separately in a shaded outdoor enclosure at CEREEP Ecotron Île-De-France (Saint-Pierre-lès-Nemours, France) in opaque 250-L drums containing continuously aerated, 50-μm-filtered lake water acquired on site (280 μS; 8.5 pH). A full water change was performed every second day within each drum. A standard diet of frozen chironomid larvae, purchased commercially, was provided ad libitum. The experimental prey, the amphipod *Echinogammarus berilloni* (TL: 0.5–0.8 cm), known to be consumed by both species in the field and in the lab (Laverty et al. 2017), and representative of the diet of both fish species, were collected from Le Lunain River, Nonville, France (48°17′24.0″N 2°47′20.6″E), via kick sampling and transported in source water to a laboratory at CEREEP Ecotron Île-De-France (19 ± 2 °C). These prey were maintained on a diet of source stream flora and fauna ad libitum in 7-L containers containing continuously aerated, filtered lake water. Fish were reused systematically in experiments following a designated recovery time (≥ 48 h) under standard diet and housing conditions (as per Alexander et al. 2015). Reuse helped minimise the number of individuals required, especially of the endangered native bullhead (see also Ethics statement).

### Individual functional responses (FRs)

We quantified FRs of both fish species at three oxygen regimes over six prey densities. Treatments were fully randomised spatially and temporally to eliminate block effects. Prior to FR experiments, fish were starved separately for 24 h in the laboratory (19 ± 2 °C; 12:12 light regime) to standardise hunger levels. Following starvation, fish were introduced individually to 7-L opaque polypropylene arenas (33.5 × 24.5 cm) containing filtered lake water and allowed to acclimatise for 2.5 h. Similarly, *E. berilloni* prey were added to 2-L arenas with filtered lake water at each of six densities (2, 4, 8, 16, 32 and 64; *n* = 3 per experimental group) and acclimatised to the experimental treatments in the same increments. The experiment was run at 19 °C under three levels of oxygen saturation: 90%, 60% and 30% (i.e. 8.4 mg L^−1^, 5.6 mg L^−1^ and 2.8 mg L^−1^ oxygen, respectively), monitored continually using a FireSting oxygen probe (Pyro Science, Germany). Oxygen was slowly reduced from 100% saturation (9.3 mg L^−1^) in each experimental arena in three 50-min increments, first to 90 ± 2%, then 60 ± 2%, and finally 30 ± 2%, by bubbling nitrogen through the water, following the method of Dick et al. (1998) and Laverty et al. (2015). While we acknowledge that adaptation was brief, we aimed to strike a balance between the stress of insufficient acclimatisation and that of keeping the fish in arenas individually with repeated disturbance. These oxygen levels were chosen to be representative of pristine through to degraded waterways (e.g. see Huang et al. 2017 which studied dissolved oxygen levels in an urban river ranging from supersaturated, 11.5 mg L^−1^, to depleted, 3.6 mg L^−1^, near a wastewater treatment plant). Disturbance in experimental arenas (bubbling and use of oxygen probe), was standardised during all incremental oxygen reductions. Trials were initiated following the addition of designated prey densities to each experimental unit, with fish allowed to feed subsequently for 1 h. Controls consisted of a replicate under each level of ‘oxygen regime’ and ‘prey density’ to account for any potential background prey mortality. Following the feeding period, fish were removed and remaining live prey counted to derive prey numbers consumed.

### Invasion-stage mesocosms

To examine the effects of invasion stage on the predatory impact of the invasive *N. melanostomus* and the native *C. gobio* across oxygen levels, we employed a factorial design with respect to ‘invasion stage’ (4 levels: “Pre-invasion”, “Arrival”, “Replacement” and “Proliferation”, see Fig. 1) and ‘oxygen regime’ (3 levels: 90%, 60% and 30%, as above) in a mesocosm experiment. Fish were starved for 24 h prior to experimentation in a shaded outdoor enclosure (14 ± 2 °C) after which they were introduced into one of four treatment combinations (Pre-invasion: 2 × *C. gobio*; Arrival: 2 × *C. gobio* + 2 × *N. melanostomus*; Replacement: 2 × *N. melanostomus*; Proliferation: 4 × *N. melanostomus*). Each arena was an opaque 50-L (80 × 63 cm) container with filtered lake water from a continuously-aerated source. Similarly, the focal prey, *E. berilloni*, were separately adapted at densities of 200 individuals in 2-L arenas containing previously aerated, filtered lake water (*n* = 3 per experimental group). Methods to reduce oxygen concentration for predators and prey were similar to those used in the FR experiment with staged declines (i.e. 90% saturation, 9.3 mg L^−1^; 60%, 6.2 mg L^−1^; and 30%, 3.1 mg L^−1^ at 14 °C), starting from total saturation (100%, 10.3 mg L^−1^), by bubbling nitrogen gas through experimental water. Trials were initiated through the addition of the 200 prey individuals to each replicate, with fish allowed to feed for 1 h. Controls comprised a replicate under each oxygen regime with no fish present. After the feeding period, fish were removed and remaining live prey counted to quantify prey numbers consumed.

### Prey activity across oxygen levels

Changes in activity levels of *E. berilloni* were measured across the three oxygen levels above to account for effects of the experimental oxygen conditions on prey activity and subsequent fish predatory rate. Prey (*n* = 9 per oxygen treatment) were slowly acclimated to the experimental oxygen saturation levels, in the same stepwise manner described earlier, before being added individually to 500 ml of water (90, 60 and 30%, ± 2% saturation; lab temperature 19 ± 2 °C) in glass crystallising dishes via pipetting. Each dish had a line drawn across the diameter of the base, and individual activity was recorded using a CX action camera (ACTIVEON Inc., U.S.A.) and watched back, with the number of line-crosses over the following 10 min counted as an indicator of *E. berilloni* movement, from the addition of individual *E. berilloni* to each dish.

### Data analyses

Our analyses followed three key steps to quantify system-level ecological impacts of invasive species under oxygen regime shifts. We first modelled functional responses of the invasive and native fishes, with a significantly negative first-order term being indicative of a destabilising Type II FR (i.e. little refuge for prey at low densities: Dick et al. 2014), whilst a significantly positive first-order term, followed by a significantly negative second-order term, is considered a stabilising Type III FR (a degree of prey refuge at low prey densities; further outlined in Supplementary Material). We also compared their attack rate (*a*) and handling time (*h*) parameters across different oxygen regimes, both visually with 95% confidence intervals and using the difference method (see Supplementary Material for further details). Second, we combined maximum feeding rates (1/*h*) from the functional responses with field-reported densities of the invasive and native fishes to quantify their Relative Impact Potential (RIP) across different oxygen regimes (see Supplementary Material). Density data for the invader were taken from Masson et al. (2018), which measured *N. melanostomus* densities at three points of the Moselle river—a long established area, an old invasion front and the current invasion front. Density data for the native *C. gobio* were recorded by *Fédération de Seine-Et-Marne Pour La Pêche et la Protection du Milieu Aquatique* in Ru du Dragon on the 4th October 2018, a river in which the invader has not yet established. As RIP qualifies impacts of invaders relative to natives, RIP scores > 1 indicate a higher ecological impact of invaders compared to natives. Thirdly, Relative Total Impact Potential (RTIP) was used to quantify and compare system-scale ecological impacts of invasive and native species, using both functional response and fish density data (see Supplementary Material) and invasion-stage mesocosm results. This allowed for comparative assessment of ecological impacts at hypothetical invasion stages based on different invader/native predator compositions. Here, similar to above, RTIP scores > 1 indicate greater ecological impacts than pre-invaded communities.

The numbers of line crosses of the prey species *E. berilloni* with respect to ‘oxygen regime’ (3 levels: 90%, 60% and 30%) were analysed using GLMs, which assumed a quasi-Poisson distribution. In each model, backward eliminations of non-significant terms and interactions resulted in the most parsimonious fits (Crawley 2014). Further details are provided in the Supplementary Material.

## Results

### Individual functional responses (FRs)

**Fig. 2:**
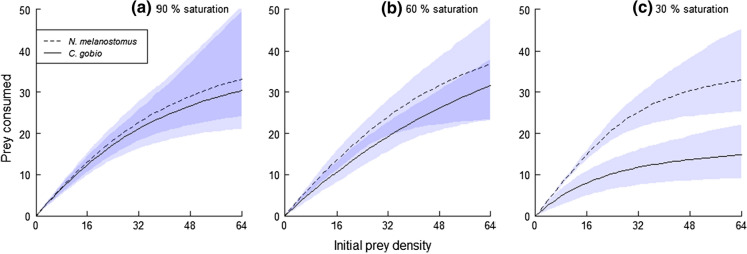
Functional Responses of *Neogobius melanostomus* (dashed line) and *Cottus gobio* (solid line) towards *E. berilloni* at 90% (a), 60% (b) and 30% (c) dissolved oxygen saturation levels. Grey areas are bootstrapped 95% confidence intervals

Survival of prey in fish-free control groups was 99.5% or higher, therefore experimental deaths were attributed to predation, which was often also observed. Destabilising Type II FRs were recorded for all predator and oxygen treatments, with significantly negative first-order terms in each instance (Table 1; Fig. 2; Supplementary Material). At 90% oxygen saturation, attack rates did not differ significantly between *N. melanostomus* and *C. gobio* (*z* = 0.53, *p* = 0.59) and neither did handling times or maximum feeding rates (*z* = 0.54, *p* = 0.59; see Table 1; Fig. 2; Supplementary Material). At 60% oxygen saturation, attack rates of the invader *N. melanostomus* were significantly higher than the native *C. gobio* (*z* = 2.64, *p* = 0.008), whereas handling times and maximum feeding rates remained similar (*z* = 0.485, *p* = 0.628; see Table 1; Fig. 2; Supplementary Material). At 30% oxygen saturation, however, attack rates of *N. melanostomus* were significantly higher than those of *C. gobio* (z = 3.98, *p* < 0.001) and handling times significantly lower, leading to significantly higher maximum feeding rates of the invader (*z* = 3.05, *p* = 0.002; Table 1; Fig. 2; Supplementary Material). This latter result is clearly demonstrated by a lack of overlap between the 95% confidence interval clouds for the two species and the native species feeding rate dropping radically at the low oxygen saturation (Fig. 2).

**Table 1:** First order terms and significance levels resulting from logistic regression of proportional prey consumption across different prey densities for the invader, *Neogobius melanostomus*, and the native, *Cottus gobio*

Predator	Oxygen (% conc.)	First order term, *p*	Attack rate (*a*), *p*	Handling time (*h*), *p*	Maximum feeding rate (1/*h*)
*Neogobius melanostomus*	90	− 0.04, < 0.001	2.30, < 0.001	0.021, < 0.001	48.90
*Cottus gobio*	90	− 0.04, < 0.001	2.07, < 0.001	0.023, < 0.001	44.24
*Neogobius melanostomus*	60	− 0.03, < 0.001	2.42, < 0.001	0.018, < 0.001	57.16
*Cottus gobio*	60	− 0.02, 0.002	1.30, < 0.001	0.015, < 0.001	66.66
*Neogobius melanostomus*	30	− 0.05, < 0.001	4.28, < 0.001	0.025, < 0.001	39.60
*Cottus gobio*	30	− 0.03, < 0.001	1.20, < 0.001	0.053, < 0.001	18.95

Parameter estimates (*a*, *h*, 1/*h*) of functional responses between species and across oxygen regimes from Rogers’ random predator equation

### Relative Impact Potential

**Fig. 3:**
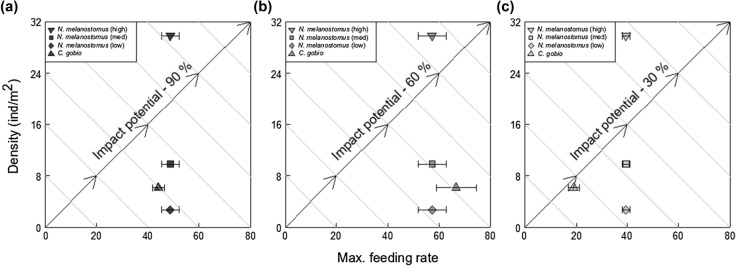
Biplots showing Relative Impact Potentials of *N. melanostomus* and *C. gobio* towards *E. berilloni* prey under 90% (a), 60% (b) and 30% (c) dissolved oxygen regimes. Each plot shows high, medium and low estimates of *N. melanostomus* density obtained from the Moselle river, France as per Masson et al. (2018), with *C. gobio* data from the uninvaded Ru du Dragon, France from a survey on the 4^th^ October 2018 by *Féderation de Seine-Et-Marne Pour La Peche et la Protection du Milieu Aquatique*. In Table 2, Relative Impact Potential (RIP) is calculated as the product of fish maximum feeding rate and fish density. In this Figure, these two measures are on the x and y axes respectively, with impact increasing along the diagonal arrows from the bottom left of the plot to the top right corner. Maximum feeding rate standard errors derived from bootstrapped data (*n* = 30)

Densities of *N. melanostomus* in the Moselle River were 2.70 ind. m^−2^ at the most recent invasion front, 9.80 ind. m^−2^ at what had been the invasion front in the prior year and 29.80 ind. m^−2^ in the long-invaded area (taken from Masson et al. 2018; see our Table 2). *C. gobio* density in Ru du Dragon was 6.21 ind. m^−2^ (*Fédération de Seine-Et-Marne Pour La Pêche et la Protection du Milieu Aquatique*; Table 2). Across all oxygen saturation treatments, ‘*N. melanostomus* (high density)’ had an RIP > 1 (Table 2, Fig. 3a–c), with the greatest RIP value at 30% oxygen saturation (10.03 at 30% v 5.31 at 90%; v 1.66 at 60%). Medium densities of *N. melanostomus* also had higher impacts than those of *C. gobio* across all oxygen treatments; however, again, this pattern was most pronounced at 30% saturation (3.30 at 30% v 1.74 at 90%; v 1.35 at 60%). Low densities of *N. melanostomus* had lower impacts than did the native fish across all oxygen treatments (i.e. RIP < 1); however, at 30% saturation, the much higher feeding rate of the invader led to a RIP score close to 1 (0.91: Table 2; Fig. 3c) using the low density estimate. This equates to half the number of invaders exerting a similar impact to the native (Fig. 3c).

**Table 2:** The Impact Potential (IP) and Relative Impact Potential (RIP) of the invasive species *N. melanostomus* relative to the native *C. gobio*, at three estimated invader densities, across three oxygen saturation levels

Species	Oxygen (% conc.)	MFR (1/*h*)	Density (ind m^−2^)	Impact Potential	Relative Impact Potential of IAS
*N. melanostomus* (low)	90	48.90	2.70	132.04	0.48
*N. melanostomus* (medium)		48.90	9.80	479.25	1.74
*N. melanostomus* (high)		48.90	29.80	1457.31	5.31
*C. gobio*		44.24	6.21	274.70	N/a
*N. melanostomus* (low)	60	57.16	2.70	154.34	0.41
*N. melanostomus* (medium)		57.16	9.80	560.20	1.35
*N. melanostomus* (high)		57.16	29.80	1703.46	1.66
*C. gobio*		66.66	6.21	413.93	N/a
*N. melanostomus* (low)	30	39.60	2.70	106.91	0.91
*N. melanostomus* (medium)		39.60	9.80	388.04	3.30
*N. melanostomus* (high)		39.60	29.80	1179.96	10.03
*C. gobio*		18.95	6.21	117.69	N/a

Impact Potential is calculated as the product of MFR (Functional Response maximum feeding rate: 1/*h*: see Table 1) and density, with the RIP of an invader calculated as the $\left( {\frac{FRinvader}{{FRnative}}} \right) \times \left( {\frac{DENinvader}{{DENnative}}} \right)$

### Relative Total Impact Potential using fish functional response and density data

**Fig. 4:**
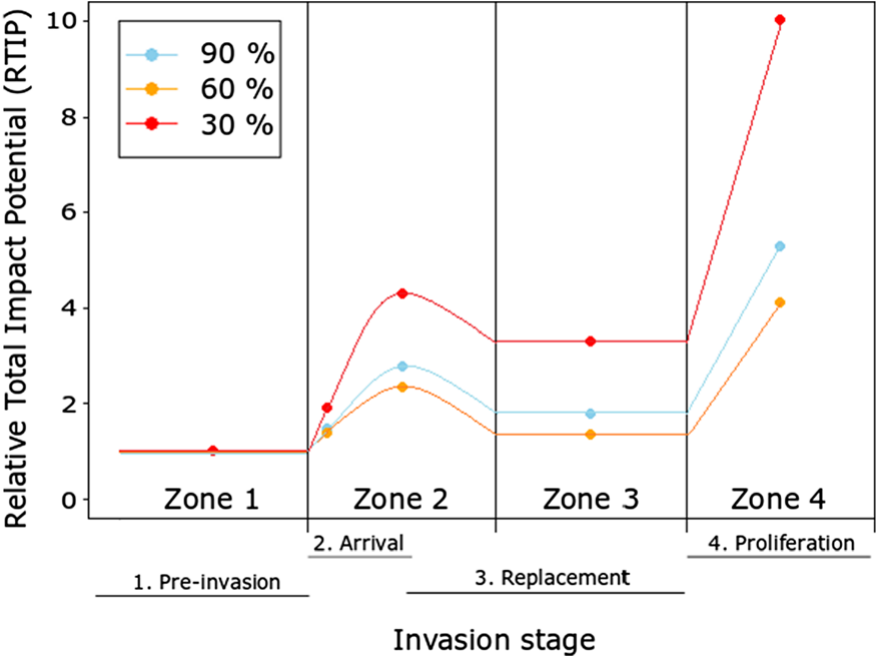
The conceptual spatio-temporal patterns of invasion impact across four invasion stages (see Fig. 1), under three oxygen saturation levels, populated by maximum feeding rate data from the FR experiments, and *N. melanostomus* and *C. gobio* field densities. Relative Total Impact Potential scores derived by comparing total impact of each invasion stage relative to that of the Pre-invasion baseline stage

Across all oxygen saturation treatments, RTIP was consistently highest in the Proliferation stage of invasion, followed by the Arrival (II) stage (Table 3; Fig. 4). RTIP scores were highest at 30% oxygen saturation levels (Table 3; Fig. 4). No RTIP scores were less than 1, indicative of heightened impacts upon the system relative to the Pre-invasion baseline (Table 3; Fig. 4) across all invader densities, stages of invasion and oxygen regime, with the low oxygen regime leading to a particularly high invader impact (Table 3; Fig. 4).

**Table 3:** Relative Total Impact Potential (RTIP) using MFR (Functional Response maximum feeding rates) derived from our CFR experiment and field density data (ind m^−1^). *Neogobius melanostomus* density across its invasion of the Moselle River, France taken from Masson et al. (2018), with *C. gobio* density data taken from the Ru du Dragon, France which has yet to be invaded by *N. melanostomus* (*Fédération de Seine-Et-Marne Pour La Pêche et la Protection du Milieu Aquatique*)

Invasion stage	Density (*N. melanostomus*)	Density (*C. gobio*)	MFR (*N. melanostomus*)	MFR (*C. gobio*)	Impact Potential (*N. melanostomus*)	Impact Potential (*C. gobio*)	Total Impact Potential	RTIP
*90%*								
1. Pre-invasion	0.00	6.21	48.90	44.24	0.00	274.70	274.70	1.00
2. Arrival (I)	2.70	6.21	48.90	44.24	132.04	274.70	406.74	1.48
3. Arrival (II)	9.80	6.21	48.90	44.24	489.05	274.70	763.75	2.78
4. Replacement	9.80	0.00	48.90	44.24	489.05	0.00	489.05	1.78
5. Proliferation	29.80	0.00	48.90	44.24	1457.31	0.00	1457.31	5.31

Invasion stage	No. *N. melanostomus*	No. *C. gobio*	MFR (*N. melanostomus*)	MFR (*C. gobio*)	Impact Potential (*N. melanostomus*)	Impact Potential (*C. gobio*)	Total Impact Potential	RTIP
*60%*								
1. Pre-invasion	0.00	6.21	57.16	66.66	0.00	413.93	413.93	1.00
2. Arrival (I)	2.70	6.21	57.16	66.66	154.34	413.93	568.27	1.37
3. Arrival (II)	9.80	6.21	57.16	66.66	560.20	413.93	974.13	2.35
4. Replacement	9.80	0.00	57.16	66.66	560.20	0.00	560.20	1.35
5. Proliferation	29.80	0.00	57.16	66.66	1703.46	0.00	1703.46	4.12

Invasion stage	No. *N. melanostomus*	No. *C. gobio*	MFR (*N. melanostomus*)	MFR (*C. gobio*)	Impact Potential (*N. melanostomus*)	Impact Potential (*C. gobio*)	Total Impact Potential	RTIP
*30%*								
1. Pre-invasion	0.00	6.21	39.60	18.95	0.00	117.69	117.69	1.00
2. Arrival (I)	2.70	6.21	39.60	18.95	106.91	117.69	224.60	1.91
3. Arrival (II)	9.80	6.21	39.60	18.95	388.04	117.69	505.73	4.30
4. Replacement	9.80	0.00	39.60	18.95	388.04	0.00	388.04	3.30
5. Proliferation	29.80	0.00	39.60	18.95	1179.96	0.00	1179.96	10.03

### Relative Total Impact Potential derived from mesocosms

**Fig. 5:**
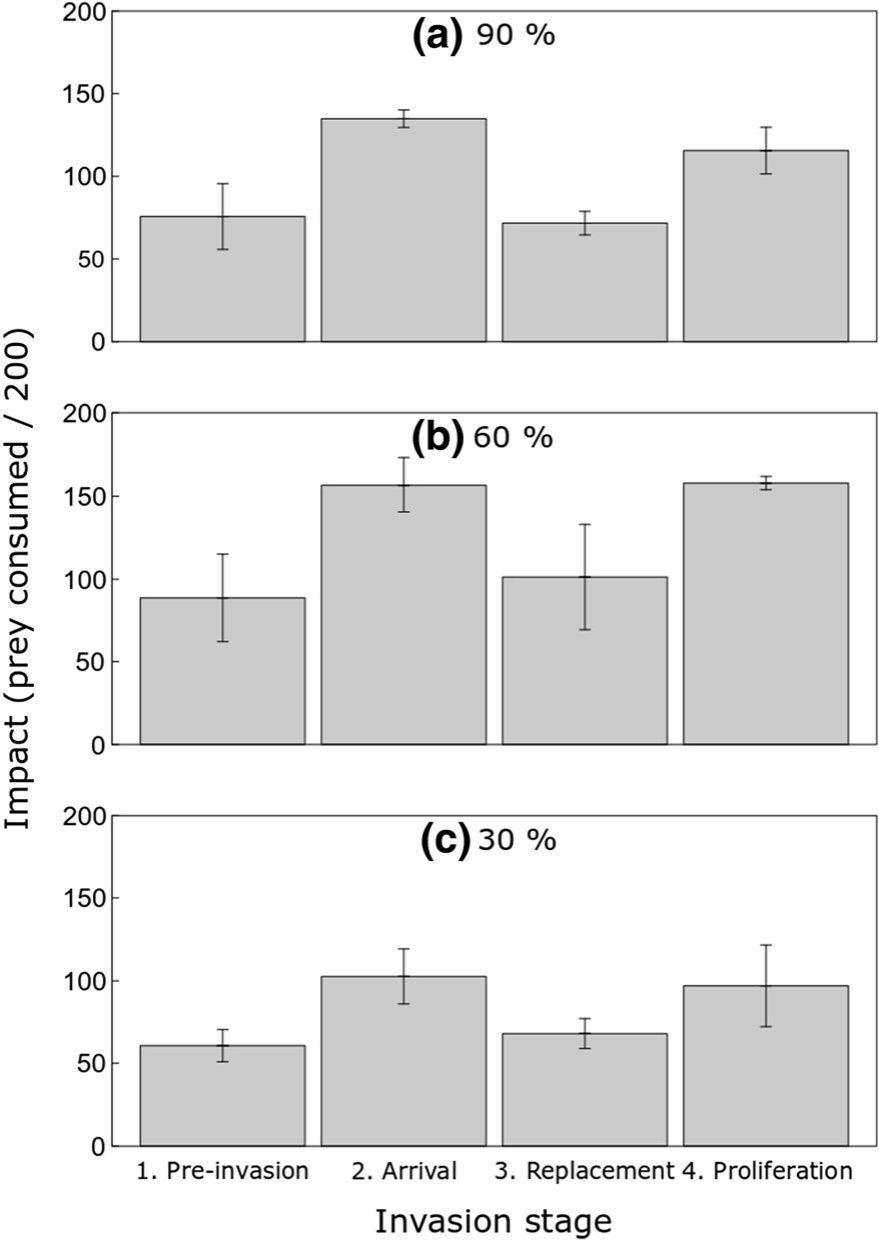
Impact exerted (total prey consumption) across invasion stages (1. Pre-invasion: 2 × *C. gobio*; 2. Arrival: 2 × *C. gobio* + 2 × *N. melanostomus*; 3. Replacement: 2 × *N. melanostomus*; 4. Proliferation: 4 × *N. melanostomus*) towards *E. berilloni* prey at 90% (a), 60% (b) and 30% (c) oxygen saturation levels. Means are ± 1 standard error

Survival of prey in control groups with no fish exceeded 99.5%, therefore prey deaths were attributed to experimental fish predation, which was again often observed directly. Prey consumption was significantly influenced by invasion stage (*F*_3, 30_ = 9.28, *p* < 0.001; Table 4; Fig. 5), with consumption significantly greater at Arrival (2 × *C. gobio*, 2 × *N. melanostomus*) and Proliferation (4 × *N. melanostomus*) stages as compared to Pre-invasion (2 × *C. gobio*) and Replacement (2 × *N. melanostomus*; all *p* < 0.01; Fig. 5). On the other hand, there were no significant consumptive differences between Pre-invasion and Replacement (*p* = 0.98) or Arrival and Proliferation (*p* = 0.931). Oxygen also had a significant influence on prey consumption overall (*F*_2, 33_ = 6.97, *p* = 0.003; Fig. 5), with consumption at 30% oxygen saturation significantly lower than at 60% saturation (*p* < 0.001), whilst consumption at 90% did not significantly differ to that at 60% or 30% oxygen saturation levels (both *p* > 0.05). The effect of oxygen regime on consumption did not change significantly (*F*_6, 24_ = 0.34, *p* = 0.91) depending on the invasion stage, owing to a non-significant ‘invasion stage × oxygen regime’ interaction effect.

**Table 4:** Relative Total Impact Potential (RTIP) with mesocosm data (using Pre-invasion stage as baseline), and predator *per capita* effect for each simulated ‘invasion stage’ across three oxygen saturation regimes (90%, 60% and 30%)

Invasion stage	No. *N. melanostomus*	No. *C. gobio*	Impact (mean consumption/200)	RTIP
*90%*				
1. Pre-invasion	0	2	75.67	1.00
2. Arrival	2	2	135.00	1.78
3. Replacement	2	0	71.67	0.95
4. Proliferation	4	0	115.67	1.53
*60%*				
1. Pre-invasion	0	2	88.67	1.00
2. Arrival	2	2	156.67	1.77
3. Replacement	2	0	101.33	1.14
4. Proliferation	4	0	158.00	1.78
*30%*				
1. Pre-invasion	0	2	60.67	1.00
2. Arrival	2	2	102.67	1.69
3. Replacement	2	0	68.00	1.12
4. Proliferation	4	0	97.00	1.60

**Fig. 6:**
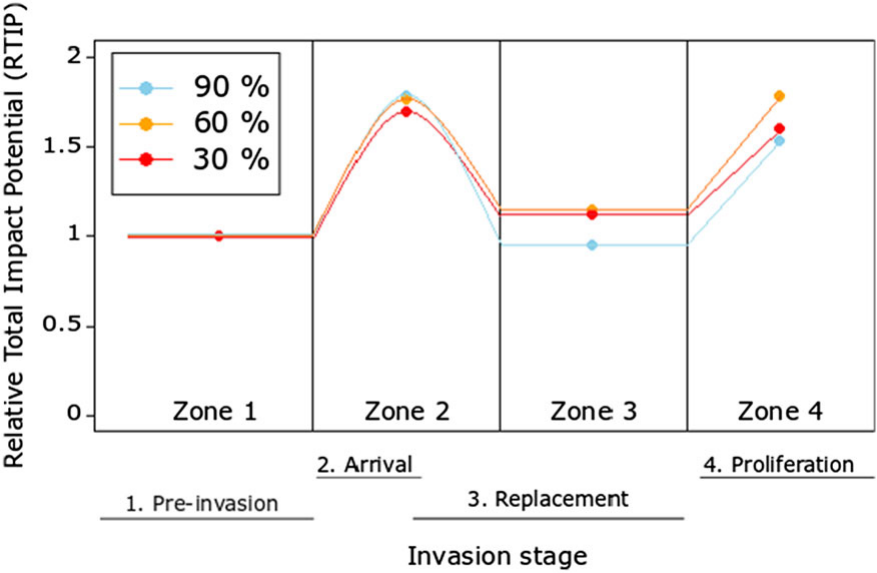
The conceptual spatio-temporal patterns of invasion impact across four invasion stages (1. Pre-invasion: 2 × *C. gobio*; 2. Arrival: 2 × *C. gobio* + 2 × *N. melanostomus*; 3. Replacement: 2 × *N. melanostomus*; 4. Proliferation: 4 × *N. melanostomus*), under three oxygen treatment levels, populated using the consumption data from our mesocosm experiment. Relative Total Impact Potential scores derived by comparing total impact of each invasion stage relative to that of the Pre-invasion baseline stage

Comparison among stages revealed that the Arrival stage, the Replacement stage and the Proliferation stage all had greater RTIP than the Pre-invasion stage, with the exception of the Replacement stage at 90% oxygen saturation (RTIP < 1), when two *N. melanostomus* exerted a lower impact upon the prey than did two native *C. gobio* (Table 4; Fig. 6). At 90% and 30% saturation, the Proliferation stage, containing four *N. melanostomus*, had a lower RTIP than the Arrival stage, which featured two *N. melanostomus* and two *C. gobio* (90%: 1.52 v 1.78; 30%: 1.60 v 1.69), with the Proliferation stage having only a slightly larger RTIP at 60% saturation (1.78 v 1.77).

### Oxygen level and *E. berilloni* activity

*Echinogammarus berilloni* activity did not differ significantly among the three oxygen regimes (*F*_2,26_ = 0.45, *p* = 0.64, Fig. S1 in Supplementary Materials). We therefore attribute differences in predator performance to variations in fish foraging abilities under different oxygen regimes.

## Discussion

Climate change and IAS are interacting on a global scale and we thus need clear forecasts of their combined effects on biodiversity in the twenty-first century (Walther et al. 2009; Johnson et al. 2017); however, such combined threats of climate change and IAS tend to be overlooked (Fey and Herren 2014). To address this gap, we quantified how three oxygen levels (as % saturation) affected: functional responses (FRs) of an IAS, the Ponto-Caspian invasive fish, *N. melanostomus*, and trophically analogous, endangered native, *C. gobio*; the impact on prey populations exerted by both fish using the Relative Impact Potential (RIP) metric; and how the combined impacts of both fish change over invasion stages (Pre-invasion, Arrival, Replacement, Proliferation) using the novel Relative Total Impact Potential (RTIP) metric. From the comparative FR (CFR) experiment, we found that the invader had a significantly higher feeding rate relative to the native at low oxygen levels (30%). RIP revealed that low oxygen exacerbates the high relative impact of the invader, while our first calculation of RTIP (based on field abundances) was consistently higher at low oxygen and especially high during invader Proliferation. In a mesocosm experiment, low oxygen lowered RTIP where both species were present, however, the IAS retained high relative impact during Replacement and Proliferation invasion stages.

The CFR method (Dick et al. 2014) demonstrated that prey consumption at 90% and 60% levels was similar between the IAS and native species, whilst the 30% treatment led to significantly reduced consumption by the native relative to the IAS. Mismatches between demand for oxygen and oxygen supply to tissues have been shown to negatively impact a number of higher functions, including muscular performance, behaviour, growth and reproduction (Pörtner and Knust 2007). Here, we found that the feeding efficiency of the IAS was more robust to declines in oxygen levels expected to arise from global environmental change (Jenny et al. 2016; Adrian-Kalchhauser et al. 2020), with significantly higher attack rates, lower handling times, and higher maximum feeding rates than the native fish at 30% saturation. *N. melanostomus* are known to be tolerant of low dissolved oxygen levels, with their blood having high oxygen affinity (Soldatov 1997). While *N. melanostomus* are thought to prefer shallow water habitats (Kornis et al. 2012) and are regularly found in areas of high oxygen saturations (Jakubčinová et al. 2018), their low oxygen tolerance may contribute to their tendency to persist at great depths in native and invaded ranges (e.g. recorded at 130 m in Lake Ontario, Canada; Walsh et al. 2007). This adaptation may also help their survival of certain ballast water treatments (e.g. Tamburri et al. 2002).

Despite no significant difference between the two species in terms of prey consumption at 90% or 60% oxygen saturation, the IAS had significantly higher attack rates at a 60% saturation, indicating a propensity to exert greater impact at low prey densities. Furthermore, an adaptation to survive low oxygen levels may facilitate effective predation at low light intensities where oxygen depletion is more common (Jenny et al. 2016). The higher performance of this Ponto-Caspian goby supports the idea that this region, due to its variable abiotic regimes, has led to the evolution of characteristics that have favoured such species as invaders in a number of novel ecosystems globally (Casties et al. 2016; Cuthbert et al. 2020).

Assessment of ecological impact with the RIP metric (RIP: Dick et al. 2017b), using FRs as above and density data from the Moselle River (*N. melanostomus* established) and Ru du Dragon (native predominates), revealed that effects of invasive *N. melanostomus* were enhanced by hypoxic conditions characteristic of warming lakes and rivers (Jenny et al. 2016). While *C. gobio* have been shown to maintain populations in polluted rivers, such as agricultural areas with elevated nutrient loads (e.g. Great Ouse catchment, England: Copp 1992; Carter et al. 2004), evidence suggests that the combination of such abiotic stressors alongside IAS can have severe detrimental effects on the native (Lorenzoni et al. 2018). Consistent with this suggestion, our RIP biplots demonstrate that, even if *C. gobio* can maintain similar densities under different oxygen levels (Legalle et al. 2005), the potential impact it can exert on the aquatic food web is highest at 60% saturation, driven by its relatively high feeding rate at this oxygen level. In contrast, an obvious disparity in predatory capability and ecological impact emerges at 30% oxygen saturation, something further supported by the mesocosm experiment (see below), with the IAS capable of exerting a similar impact to the native at half the density.

Although we assessed *per capita* feeding rates at differing predator densities, we have not assessed the direct effect of declining dissolved oxygen levels on the relative densities (or other appropriate proxies of numerical response, e.g. fecundity) of the two species. However, Jones and Reynolds (1999) found no decrease in hatching success as a function of oxygen levels for common gobies (*Pomatoschistus microps*), possibly because they exhibit adaptive behaviours that compensate for decreased oxygen levels, such as increased time spent fanning their eggs (Jones and Reynolds 1999). Another behavioural adaptation is shown in sand gobies (*Pomatoschistus minutus*), which construct their nests to enhance oxygen flow (Lissåker et al. 2003). Therefore, while we lack studies on the effect of decreased oxygen levels on the nest-guarding behaviour of our study species, comparisons with other gobies suggest that the challenges posed by decreasing dissolved oxygen levels may be surmountable above a certain threshold. Our CFR experiments highlighting *N. melanostomus* as a more effective forager at 30% saturation relative to *C. gobio* may also indicate that other energetically-costly activities (e.g. egg fanning) could prove more difficult for *C. gobio* at low oxygen saturation levels found in warming waters (Jenny et al. 2016), thus potentially leading to declines in recruitment and population size. Nevertheless, the tendency for *N. melanostomus* to move away from hypoxic conditions, when possible, should not be discounted (Arend et al. 2011).

Our two measures of RTIP were used to demonstrate temporal shifts in ecological impact over different invasion stages of IAS arrival and establishment. First, we simulated these stages of invasion (we divided ‘Arrival’ into two parts to incorporate the spatio-temporal changes in *N. melanostomus* density as per Masson et al. 2018) by combining our FR maximum feeding rate data (across the three oxygen treatments) with fish density data from the field. Due to the high density of *N. melanostomus* in the long-established region of the Moselle river, we saw the Proliferation stage having the highest RTIP score across all oxygen treatments. With no RTIP scores <1 post-invasion, we can attribute heightened impact upon the system as a result of the IAS. The highest RTIP scores for each invasion stage were found at the 30% treatment, but the lowest were found under the 60% treatment, highlighting the importance of potential synergies between abiotic stressors and the impacts of invasive species.

Application of this method of RTIP, comprising individual FRs, assumes linear increases in potential impact with the density of the invasive species. This may fail to account for potential intra- and interspecific synergies or antagonisms between multiple consumers in a natural food web i.e. Multiple Predator Effects or MPEs (see Griffen and Delaney 1980; Médoc et al. 2013). We addressed this issue by assessing MPEs within a mesocosm setting, and again simulated four invasion stages under the three oxygen saturation treatments. We showed clearly that oxygen saturation treatment significantly influenced overall prey consumption. Across all three oxygen treatments, consumption was significantly greater in the Arrival and Proliferation stages, versus the Pre-invasion and Replacement stages, although this is to be expected considering the presence of four predators present in the Arrival and Proliferation stages versus two in the Pre-invasion and Replacement stages. However, it is feasible that multiple predators are so antagonistic as to actually lower *per capita* and even overall predation effects (Mofu et al. 2019). Arrival, Replacement and Proliferation stages had RTIP scores > 1 (i.e. indicating increased impact) relative to the Pre-arrival baseline, with the exception of the Replacement stage at 90% saturation. This corroborates with the pattern shown in our CFR experiment, with *C. gobio* shown to have a similar feeding rate to *N. melanostomus* at 90% and 60% saturation, but a significantly lower feeding rate at 30%. Also consistent with our CFR experiment, predatory impact tended to be greater for both IAS and native species at 60%, suggesting that highly oxygenated water is not optimal for either species. With both focal fish being benthic species, it may simply be that that oxygen levels of ~ 60% are closer to those that they are accustomed to, with diurnal variation common in rivers naturally due to variation of reaction rate with temperature, and variation of photosynthesis and respiration (Shi et al. 2003).

In the mesocosm experiments, ecological impacts of the IAS on the system were unexpectedly greater in the presence of the native fish (i.e. Arrival) than in its absence (i.e. Proliferation) at 90% and 30% saturation, with the Proliferation stage having only a slightly larger RTIP at 60% saturation. While Arrival and Proliferation stages had higher RTIP, the rates of consumption were lower than might be expected based on the stages with two consumers instead of four i.e. Pre-invasion and Replacement. This could be due to the non-replacement of prey in the experiment, or indicative of agonistic interactions potentially caused by inter- and intraspecific competition. While we do not have the specific *per capita* effect of each individual in the mesocosms (but see Mofu et al. 2019 for potential methodology), our results are consistent with the findings of Kornis et al. (2014) which assessed inter- and intraspecific interactions for *N. melanostomus*, finding that *N. melanostomus* body weights decreased most when alongside high densities of conspecifics (such as our Proliferation stage versus Replacement stage), whereas natives decreased most at intermediate *N. melanostomus* densities (such as our Arrival stage). While care must be taken when applying these results to the real world, they highlight a need for spatio-temporal monitoring of IAS arrivals, and suggest that although total replacement of natives by IAS may attract the most headlines, similarly high ecological impacts to a system could occur when invasive and native trophic analogues are still coexisting. While the *C. gobio* used in this study were from a river as yet uninvaded by *N. melanostomus*, it is possible that interactions between the two species, i.e. during the Arrival stage, may differ with the loss of naiveté on the part of the native species, and we highlight this as an avenue for further study. Future studies could also implement more dissolved oxygen treatments (e.g. to assess where the “tipping point” for *C. gobio* between 60 and 30% lies), acute versus chronic dissolved oxygen treatments, and more combinations of the two species via more intermediate hypothetical invasion stages—perhaps in a similar vein to a prey switching experiment but with different ratios of predators instead of prey (Cuthbert et al. 2018; Joyce et al. 2019; McCard et al. 2021).

While the focus of this study was on the overall ecological impacts of the invasive relative to the native fish species, our methodology and results have implications for inter-specific competiton between the round goby and its native counterpart. While the use of FRs in competition theory and empiricism is debated (Dick et al. 2017a; Dickey et al. 2020), such data alongside measures of changes in competitor fitness (e.g. growth, reproduction) that flow from reduced feeding opportunities of natives, due to prey depletion by invaders, merits further attention. Indeed, much competiton theory in plants stems from “functional resource-utilization responses”, but functional responses as reported for predatory animals may offer less application to competition studies, for example, due to prey switching propensities of animals that may lessen competitive effects (Dickey et al. 2020). We encourage more integration of ecological impact i.e. FR, RIP and RTIP metrics with competition theory and empiricism to fully predict IAS impacts, especially under climate change.

Overall, this study shows that the aquatic oxygen depletion characteristic of global warming can promote higher invader feeding rates relative to those of the native, with high densities of the invader, as found in the Moselle, compounding its higher FRs, and therefore potentially enhancing its ecological impact. While there may be antagonistic interactions among *N. melanostomus* individuals, such a possibility is clearly offset by high feeding rates of individuals, greater densities and also larger body size compared to natives (Kornis et al. 2014). Fortunately, unlike many aspects of climate change, dissolved oxygen levels in lakes and rivers can be improved via direct intervention. Artificial oxygenation methods, such as constructed wetlands, are capable of increasing saturated oxygen levels as well as facilitating the removal of pollutants from water (Dong et al. 2012). Similarly, replenishment of oxygen in bottom waters through enhanced vertical circulation has been proposed for lakes (Dunalska and Wiśniewski 2016), while artificial oxygenation has been shown to be effective for some lotic ecosystems (Larsen et al. 2019). Such management interventions may prove crucial to maintaining current native populations, while resisting the establishment and ecological impacts of IAS.

## Supplementary Information

Below is the link to the electronic supplementary material.

Supplementary file1 (XLSX 19 kb)

Supplementary file2 (DOCX 998 kb)

## Data Availability

The data that support the findings of this study are available from the corresponding author upon reasonable request.

## Data availability

Underlying raw data will be made available in the Supplementary Material.
