# Supplementary material for: Breathing space: deoxygenation of aquatic environments can drive differential ecological impacts across biological invasion stages
Source: Biol Invasions. 2021 Apr 30;23(9):2831–47. doi: 10.1007/s10530-021-02542-3 (PMC8550720; doi:10.1007/s10530-021-02542-3)

**Supplementary Information for Methods**

**Data analyses**

Functional Responses (FR) were modelled using the ‘frair’ package (Pritchard *et al*. 2017). Generalised linear models with a binomial error distribution were used to infer FR types (Juliano, 2001). Here, a significantly negative first-order term is indicative of a Type II FR, whilst a significantly positive first-order term, followed by a significantly negative second-order term, is considered a Type III FR. As prey were not replaced as they were consumed, Rogers’ random predator equation was used to model FRs (Rogers, 1972):

$N_{e}=N_{0}(1-exp(a\left( N_{e}h-T \right)))$ (1)

where *N_e_* is the number of prey eaten, *N*_0_ is the initial density of prey, *a* is the attack constant, *h* is the handling time and *T* is the total experimental period. Maximum feeding rates (1/*h*) were calculated under each treatment group. The *Lambert W* function was used to solve the random-predator equation (Bolker, 2008). Non-parametric bootstraps (*n* = 2000) were used to generate 95% confidence intervals around FR curves. Following Juliano (2001), the indicator variables were used to compare FR parameters between predators at each oxygen level (Pritchard *et al.* 2017). Here, there was no adjustment of *α* for multiplicity as tests were paired across discrete oxygen levels between predator types.

The “Impact Potential” (IP) of the IAS and native species was represented as the product of FR (i.e. maximum feeding rates derived from the CFR experiment) and a proxy of NR, usually abundance or density (Dick *et al*. 2017b):

$IP=FR\times{NR}_{proxy}.$ (2)

These Impact Potentials were then combined, giving the “Relative Impact Potential” (hereafter RIP):

$RIP=\left( \frac{FRinvader}{FRnative} \right)$ $\times\left( \frac{NRproxy of invader}{NRproxy of native} \right)$ (3)

This formulation succinctly summarises the ecological impact of an invader as a single number, with: RIP scores > 1 indicating a higher ecological impact of invader compared to native; scores = 1 indicating equivalent impact; and scores < 1 indicating a lower invader impact. Density data for *N. melanostomus* were obtained from Masson *et al.* (2018), with *Cottus gobio* density data from *Fédération de Seine-Et-Marne Pour La P*ê*che et la Protection du Milieu Aquatique* (FSPPMA). RIP biplots were created using these density data and the FR mean estimated maximum feeding rates (± SE) at the three different oxygen treatments following non-parametric bootstrapping (*n* = 30).

Maximum feeding rates and density values were used to calculate Relative Total Impact Potential (RTIP), a new metric designed to assess the system-scale ecological impacts at different invasion stages relative to a pre-invasion baseline (Fig. 1; see also Dickey *et al*. 2020). Here, at any point post-invasion, the impact exerted can be assessed relative to Pre-invasion levels (t0), as follows:

$RTIP=\left( \frac{\left( Native impact t1 \right)+(Invader impact t1)}{(Native impact t0)} \right)$ (4)

$RTIP=\left( \frac{\left( FRnative t1 \times DENnative t1 \right) + \left( FRinvader t1 \times DENinvader t1 \right)}{(FRnative t0 \times DENnative t0)} \right)$ , (5)

whereby FR is the maximum feeding rate, and DEN is the density of each species. Density estimates were obtained as above from Masson *et al*. (2018) and FSPPMA, and were used to model five invasion stages (Pre-invasion, Arrival (I), Arrival (II), Replacement and Proliferation). Here we divided ‘Arrival’ into two parts to incorporate *N. melanostomus* density at the recent invasion front (Arrival I) and at the invasion front the year prior (Arrival II) as per Masson *et al*. 2018.

The mesocosm experiment provided a second measure of RTIP, over four invasion stages (Pre-invasion, Arrival, Replacement and Proliferation). Generalised linear models with a quasi-binomial error distribution were also used to analyse raw prey consumption data in the mesocosm experiment with respect to ‘invasion stage’ (4 levels) and ‘oxygen’ (3 levels). Overall prey consumption for each invasion stage led to a simplification of the RTIP equation as:

$RTIP =\left( \frac{Prey consumption t1}{Prey consumption t0} \right)$ (6)

For both measures of RTIP (eq. 5 and 6), we propose that invasion stages with RTIP > 1 have a higher ecological impact being exerted upon the system than in the Pre-invasion baseline stage, whereas stages with RTIP < 1 have a lower ecological impact being exerted. In this formulation we do not assess the individual feeding rates of each fish, but this design gives indications of multiple predator effects (MPEs), i.e. additive, synergistic or antagonistic interactions among individuals.

The numbers of line crosses of the prey species *E. berilloni* with respect to ‘oxygen regime’ (3 levels) were analysed using GLMs, which assumed a quasi-Poisson distribution. In each model, backward eliminations of non-significant terms and interactions resulted in the most parsimonious fits (Crawley, 2014). In all GLMs, analyses of deviance using Type I sums of squares were applied to report effect sizes as data were orthogonal. Tukey tests were used to perform pairwise comparisons post hoc using the ‘lsmeans’ package (Lenth, 2016). The statistical software R (v3.4.4) was used to perform statistical analyses (R Core Development Team, 2018).

**Figure S1** Activity levels of the prey species, *Echinogammarus berilloni,* at the three dissolved oxygen saturation levels: 90 % (a), 60 % (b) and 30 % (c), assessed by the number of line crosses in a crystallising dish. Means are ± 1 SE**.**


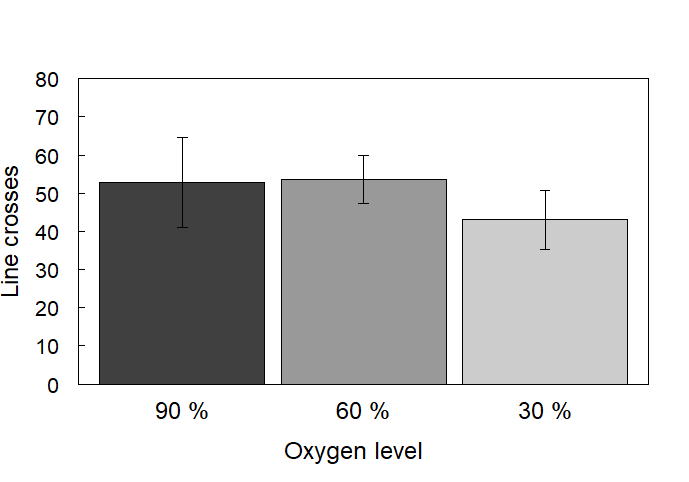

Supplement: Supplementary file 2 — Supplementary file2 (DOCX 998 kb) [file 10530_2021_2542_MOESM2_ESM.docx]
